# An anomalous course of the radial artery: Dissect rather than resect

**DOI:** 10.4103/0970-0358.73474

**Published:** 2010

**Authors:** Berkan Mersa, Bülent Özçelik, Samet Vasfi Kuvat, Özgür Pilanci

**Affiliations:** Department of Hand Surgery, İst-El Microsurgery and Rehabilitation Group, Gaziosmanpaşa Hospital, İstanbul, Turkey; 1 Department of Plastic and Reconstructive Surgery, Dicle and İstanbul Faculty of Medicine, Diyarbakir and İstanbul, Turkey; 2 Department of Plastic and Reconstructive Surgery, Bağcilar Training and Research Hospital, İstanbul, Turkey

Sir,

Carpal tunnel syndrome is the most common entrapment neuropathy. Rare aberrant-bifid tendon, muscle, nerve or arterial anomalies may be the cause of this syndrome.[1] Experienced surgeons do not fail to examine the tunnel for anomalies during the operation. We however came across an extra-tunnel arterial anomaly during one such carpal tunnel decompression.

**Figure 1:**
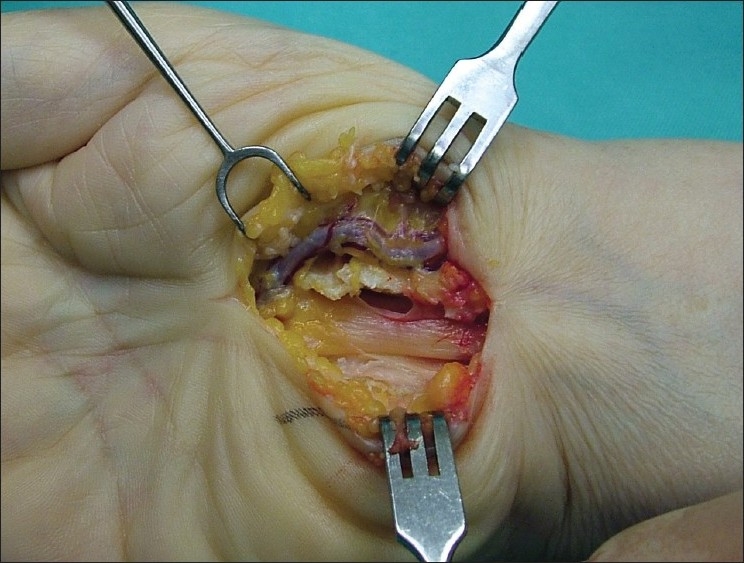
Intra-operative photograph showing the radial artery passing superficial to the transverse carpal ligament

Carpal tunnel syndrome, bilateral, albeit more severe on the right side, was diagnosed by neurophysiological examinations in a 52-year-old female patient who presented with nocturnal pain, tingling and numbness for the past 2 years. During the carpal release operation performed under axillary anaesthesia with the pneumatic tourniquet, we observed that the radial artery ran superficial to the transverse carpal ligament and turned medially distal to the ligament [Figure 1]. The distal course of the artery could not be visualised due to the limitation of the incision. Intra-tunnel structures appeared normal when the transverse carpal ligament was incised. The advanced dissection could not be done due to the mediolegal problems. After release of the pneumatic tourniquet, the abnormal vascular anatomy was confirmed with examination of the arterial pulse. The arterial pulse was dislocated medially over the snuff box. On the contralateral side, the arterial pulsation was established at the normal anatomical location. The patient refused further investigation such as angiography for complete anatomical course of the artery.

The frequency of radial artery anomalies is about 1%.[2] Publications on the radial artery anomalies are usually related to upper level arterial anomalies encountered during dissections carried out for coronary angioplasty or flap surgery.[3] Radial artery anomalies within the carpal tunnel usually present themselves with entrapment neuropathy. These very rare anomalies are usually diagnosed during decompression surgery[1]. However, the anomaly that is presented here was an extra-tunnel anomaly and it is unlikely that an arterial anomaly over the carpal ligament could trigger entrapment neuropathy.

During surgery for scaphoid non-union, Afshar[2] noted an anomalous radial artery that crossed the tendon of the flexor carpi radialis muscle and travelled superficial to the thenar muscles. The anomaly in the present case was similar to this one but it was more on the medial aspect.

Some surgeons prefer local anaesthesia during carpal tunnel decompression.[4] However, in the presence of the anomaly we encountered, infiltration of local anaesthetic agent at the operating site may result in arterial damage.

In conclusion, awareness on the possibility of encountering known intra-tunnel anomalies as well as extra-tunnel anomalies and avoiding local anaesthesia, under the light of these findings, during decompression surgery is important.
